# Effects of Long-Term Exposure to High Altitude Hypoxia on Cognitive Function and Its Mechanism: A Narrative Review

**DOI:** 10.3390/brainsci12060808

**Published:** 2022-06-20

**Authors:** Yuan Li, Yan Wang

**Affiliations:** 1 Center on Aging Psychology, CAS Key Laboratory of Mental Health, Institute of Psychology, Beijing 100101, China; charleslilovepeace@163.com; 2 Plateau Brain Science Research Center, Tibet University/South China Normal University, Lhasa 850012, China; 3 Department of Psychology, University of Chinese Academy of Sciences, Beijing 100049, China

**Keywords:** high altitude, long-term exposure, lowlanders, cognitive function, mechanism

## Abstract

Cognitive function is affected by low pressure and hypoxia in high-altitude environments, and is regulated by altitude and exposure time. With the economic development in the Qinghai-Tibet Plateau, the increase in work and study activities, as well as the development of plateau tourism, mountaineering, and other activities, the number of plateau immigrants is increasing daily. Long-term hypoxia challenges human physical and mental health, restricts work efficiency, and thus affects plateau economic development and human wellbeing. Therefore, it is of scientific and social significance to study how long-term exposure to the hypoxic plateau environment affects the physical and mental health of lowlanders as part of the ongoing development of the current plateau region. In this paper, we reviewed the research progress and mechanism of the effects of long-term (≥1 year) high-altitude (>2500 m) hypoxia exposure on the cognitive function of lowlanders, and suggested that the scope and sample size of the research should be expanded in the future, and that follow-up studies should be carried out to explore the time threshold of cognitive impairment and its compensatory or repair mechanism.

## 1. Introduction

The Qinghai diagnostic criteria for Chronic Mountain Sickness (CMS) defines the area above 2500 m as a plateau environment [1]. Many factors, such as low oxygen, low pressure, low temperature, low humidity, and high solar radiation, directly and persistently affect human health in plateau environments [2]. With the development of economic construction and the increase in activities such as plateau tourism and mountaineering, increasing numbers of people work and live in high-altitude areas, and the physical and mental health challenges posed by the plateau environment are becoming increasingly prominent [3]. Therefore, it is increasingly important to study the impact of the hypoxia environment on lowlanders’ cognitive function.

The effect of plateau environments on physical and mental state change dynamically with altitude and residence time. Zubieta-Calleja divides the physiological adaptation process into three stages, acute adaptation, subacute adaptation, and chronic adaptation, according to the changes of human hematocrit. The formula of altitude adaptation with time and space was proposed as “high-altitude adaptation factor = exposure time (day)/altitude (km)” [4]. When the altitude is 3600 m, the adaptation to the plateau changes with the time spent residing there. The first stage is the acute adaptation period (within 3 days of entering the plateau), the second stage is the subacute adaptation period (3 days after entering the plateau), and the third stage is the chronic adaptation period (1 month after entering the plateau) [4]. Accordingly, cognitive function changes with the change of physiological adaptation to the altitude. The decline of cognitive function was most obvious upon the subjects first entering the plateau. With prolonged exposure time, high altitude hypoxic acclimation occurred, and cognitive function recovered somewhat, but it was still difficult to reach the cognitive level of subjects in the plain control group [2,5]. As the altitude rises, the adaptation time to the plateau also extends correspondingly, and the higher the altitude, the more serious the impact of hypoxia on physiology, and the more obvious the decline in working ability [6]. Previous studies revealed that cognitive function decreased first, then increased, and finally decreased with the increase of altitude exposure time, and cognitive function decreased with the increase of altitude [3,7].

Early studies on the effects of high-altitude environments on cognitive function focused on the effects of acute hypoxia exposure on cognitive function, using hypobaric oxygen chambers to simulate high altitude environments, or in activities such as high-altitude mountaineering [8,9]. In terms of research subjects, attention was primarily given to special groups such as soldiers and mountaineers on the plateau, and less to the lowlanders living in the plateau area [10,11]. In terms of research methods, early studies mainly recorded responses to various psychological tasks, or adopted traditional psychophysical methods and neuropsychological tests [12]. With the development of cognitive neuroscience research techniques such as event related potentials (ERP) and functional magnetic resonance imaging (fMRI), researchers have begun to explore the brain mechanisms underlying the effects of long-term high-altitude hypoxia exposure on cognitive function [13,14,15,16,17,18]. Previous studies on long-term plateau environmental hypoxia mainly focused on the following two groups of people [19,20]. The first type is the lowlanders who grew up in the plain and went to work and live in the plateau when they grew up. The other is the highlanders who have lived on the plateau for generations, mainly Tibetans. After a long period of natural selection, highlanders have a different gene expression, physiological structure, and psychological function to people in plain areas, and are more adaptable to the plateau environment [15]. The research results of highlanders cannot be extended to lowlanders. This review focuses on the effects of long-term high-altitude exposure on the cognitive function of lowlanders.

This paper reviewed studies on the effects of long-term exposure to high-altitude environments on cognitive function of lowlanders and the underlying physiological mechanisms, in order to provide a new direction for the effects of long-term exposure to high-altitude environment on cognitive function, and to provide a scientific basis for the assessment and protection of the cognitive function of lowlanders.

## 2. Effects of Long-Term Exposure to High Altitude Hypoxia on Cognitive Function

### 2.1. Brain Structure and Brain Function Basis of Long-Term High-Altitude Exposure Affecting Cognitive Function

In previous neuroimaging studies, magnetic resonance imaging (MRI) was mostly used to explore the effects of long-term hypoxic exposure on brain structure and function.

In terms of brain structure, MRI-T1 sequence was used to collect image data, and voxel-based morphometry (VBM) analysis and measurement were performed, so as to infer the effects of long-term hypoxic exposure on gray matter, white matter, and cerebrospinal fluid in brain tissue. Adaptive changes in brain structure have been found after long-term hypoxic exposure. After two years of migration to the plateau, the thickness of the right posterior central gyrus and right superior frontal gyrus decreased significantly, while the thickness of the right middle frontal gyrus, parahippocampal gyrus, right anterior middle temporal lobe, bilateral anterior ventral pons, and right cerebellar cortex increased [21]. The thickness of bilateral insula, right anterior cingulate gyrus, bilateral prefrontal cortex, left anterior central cortex, and right lingual cortex was significantly reduced in long-term migrants [22]. The above studies suggest that when hypoxic exposure time is longer, adaptive changes occur in brain tissues, such as local cerebral vascular hyperplasia and increase in cortical thickness in local brain areas, to compensate for inadequate blood oxygen levels. With the prolonged hypoxic exposure time, the whole brain gray matter showed a tendency of atrophy, showing the characteristics of non-specific injury [21,22].

In addition, diffusion tensor imaging (DTI) was used in previous studies to analyze the change of fractional anisotropy (FA) in white matter in patients with long-term hypoxic exposure [22,23]. The results are similar to gray matter, and FA changes appear in the whole brain white matter. The FA of the corpus callosum, radiative corona, anterior longitudinal tract, and bilateral hippocampus decreased in people who migrated to the plateau for two years, while the FA of the upper and lower longitudinal tract, corpus callosum, corticospinal tract, and cortical brainstem tract increased [22]. In comparison, FA increased in the bilateral upper and lower longitudinal tracts, corpus callosum, radiative corona, posterior cingulate gyrus, and corticospinal tract in the highland immigrants of 3–4 generations. FA decreased in optic tract and upper longitudinal tract [22]. The study also analyzed the correlation between the changes of gray matter volume, white matter FA, and other parameters in the plateau population with physiological parameters and neuropsychological test results. The study found that the reduction of gray matter volume in the parahippocampus and middle frontal gyrus of the plateau population was positively correlated with the change in vital capacity, the change in the gray matter volume of the superior frontal gyrus was correlated with the outcome of the mental rotation task, and the change in the thickness of the postcentral gyrus cortex was correlated with the working memory reaction time [23]. These results suggest that long-term high altitude hypoxia exposure leads to structural changes in the whole brain, and such changes may be the structural basis of cognitive function changes.

Functional magnetic resonance imaging (fMRI) and event related potentials (ERP) were used to investigate the effects of long-term hypoxia exposure on brain function. Regional homoho (ReHo) analysis of resting state brain function of migrants shows that there is a significant increase of ReHo in the right lower sensorimotor cortex, which is correlated with the response time of memory search task. Voxel-mirrored homotopic connectivity (VMHC) analysis showed that bilateral visual cortex signals were significantly enhanced and correlated with subjects’ hemoglobin concentration, suggesting that long-term hypoxia exposure may affect the synchronization and connectivity of spontaneous brain neural activity. This may be the brain function basis for cognitive function changes [24]. ERP studies have found that long-term hypoxia affects ERP components in the parietal occipital lobe, anterior cingulate cortex, prefrontal lobe, temporal lobe, and other brain regions in the lowlanders at high-altitude areas, showing impairment of attention function, inhibitory control function, working memory function, and other cognitive functions [13,14,15,19].

### 2.2. Attention

Attention refers to the orientation and concentration of psychological activities or consciousness on certain objects [25]. It uses limited cognitive resources to process target-related information [26], and is an important psychological attribute for the generation and carrying out of all psychological processes.

Long-term hypoxia damages attention function and produces adaptive compensation in the brain. Early behavioral studies found that long-term post (15 months) had more severe damage to attention span than short-term post (3 months) [27]. Long-term high-altitude exposure lengthens the visual spatial attention response time of migrants [13,15], decreased alertness and executive control ability in attention network test (ANT) [28]. An fMRI study found that decreased gray matter volume in attention-related brain regions, such as bilateral prefrontal lobe and right cingulate gyrus, may be the neural basis of attentional impairment in Han Chinese living in high-altitude areas [18].

Event-related potentials (ERPs) are special brain-evoked potentials that use brain potentials evoked by multiple or diverse stimuli by intentionally giving stimuli special psychological meanings [29]. It reflects changes in the neuroelectrophysiology of the brain during cognitive processes, also known as cognitive potentials [30].

Using ERP technology, Wang et al. found that the P3 component amplitude of undergraduates who grew up in low-altitude areas and moved to high-altitude areas of 3650 m altitude for 3 years under high perceptual load decreased, indicating that their spatial attention function decreased [13]. In electrophysiological research, the P3 component of the parietal lobe is a typical indicator of attention maintenance, and it is also one of the important components of ERP experiments in hypoxic environments. At the same time, its N1 component (the N1 potential is located in the occipital region and is a unique component of spatial attention evoked potentials) was activated in bilateral occipital lobes, and the lateralization effect of spatial attention processing disappeared, reflecting adaptive compensation in a long-term hypoxic environment [13]. In the visual search task, Zhang et al. (2018) found that when the target appears in the right visual field, the amplitude of N2pc (N2-posterior-contralateral) components in the plateau migration group is smaller than that in the plain control group. In visual search tasks, the N2pc component is an effective electrophysiological indicator for the assignment of visuospatial attention to targets. The amplitude of the N2cc (N2-central-contralateral) component in the plateau migration group was greater than that in the plain control group. The N2cc component in the visual search task reflects blocking of cross-talk between attentional orientation and response selection [15]. In addition, the peak value of MP (Motor Potential, MP appears in the contralateral motor cortex and represent a specific response to the muscle movement state) and the latency of RAP (Reafferent Potential, RAP reflects sensorimotor integration processes) in the plateau migration group was larger than those in the plain control group [15]. The results showed that long-term hypoxia not only reduced the function of attentional resource allocation to target, but also decreased the function of target selection and response preparation.

### 2.3. Executive Function

Executive function refers to the advanced cognitive process that controls and adjusts other cognitive processes when completing complex cognitive tasks, and its fundamental role is to produce coordinated, orderly, and goal-oriented behaviors [31]. Executive function includes working memory, inhibitory control, and task switching [32]. Previous studies on the effect of high-altitude on executive control mainly focused on working memory and response inhibition.

Working memory is a mechanism for temporary processing and storage of information [33]. A behavioral study using n-back task found that, compared with the plain control group, the plateau migration group with long-term hypoxia had lower accuracy, longer response time, and slower working memory processing speed in the 2-back condition [19]. Some fMRI studies have shown that long-term hypoxia may lead to functional changes in brain regions associated with working memory [16,17,18]. The results showed that the activation of the left pyramidal and left superior temporal gyrus was greater, while the activation of left middle occipital gyrus was lesser [16,17,18]. The activation intensity of brain regions related to speech working memory, such as inferior frontal gyrus, middle frontal gyrus, occipital middle lobe, and cerebellum decreased significantly. The P2 component belongs to the early component of working memory, which reflects the allocation of attention resources of working memory in the coding stage [34]. The LPP component belongs to the late component of working memory, reflecting the allocation of attentional resources in the matching stage of working memory [35]. Compared with the plain control group, the P2 amplitude was more positive and the late positive potential (LPP) amplitude was more negative in the plateau migration group under 2-back condition [19]. The early delta band (1–4 Hz,160–300 ms) has higher energy values, while the late Delta band (1–4 Hz, 450–650 ms) and Theta band (4–8 Hz, 450–650 ms) have lower energy values [19]. The results showed that the attentional resources input of the plateau migration group decreased in the late matching stage of working memory processing, resulting in impaired response inhibition ability and information maintenance ability, and impaired spatial working memory ability [19].

Response inhibition refers to the ability to inhibit inappropriate behaviors that do not meet current needs, which is crucial for people to make flexible and goal-directed behaviors based on environmental changes [36]. Neuroimaging studies have found that long-term exposure to high altitude results in decreased gray matter volume and white matter quality in the bilateral prefrontal lobe, bilateral anterior insula, right cingulate gyrus, left anterior central gyrus, right lingual gyrus, and occipital cortex. The number of neurons decreased [17,18], and these brain regions are important regions involved in the response inhibition function. Using ERP technology, scholars studied the inhibition and control ability of college students residing at high-altitude areas for more than two years. In the Flanker task, compared with the plain control group, P3 amplitude (the P3 is an index of conflict resolution) in the plateau migration group was lesser under inconsistent conditions, indicating that the plateau migration group needed to invest more cognitive resources to resolve conflict with the same task difficulty [14]. In the Go/No-Go task, compared with the plain control group, the latency of NoGo-N2 component (the NoGo-N2 component embodies the conflict monitoring process during the early processing stage of response inhibition, and its latency reflects the processing speed of this process) was prolonged in the plateau migration group, indicating that prolonged residence at the plateau affected the individual’s conflict processing speed, and the plateau migration group showed excessive activation of neural activity in the response examination and response monitoring stage [37,38]. However, none of the above studies found the effect of long-term high-altitude exposure on response inhibition at the behavioral level. The researchers believe that because the experimental paradigm is too simple, future studies using relatively complex inhibition tasks may reveal the effect of long-term high-altitude exposure at the behavioral level.

## 3. Physiological Mechanism of Long-Term High-Altitude Hypoxia Environment Affecting Cognitive Function

The effect of high altitude and low-oxygen environment on cognitive function is due to the change of physiological mechanism. The researchers explored the relationship between high-altitude hypoxia environment and physical and mental function from the three levels of stress mechanism, cellular mechanism, and molecular mechanism.

### 3.1. Stress Mechanism

Stress is a series of physiological and psychological reactions produced by the body to maintain homeostasis balance when homeostasis is threatened [39]. Hypoxia, as the primary plateau stress source, leads to high-altitude hypoxia stress, and physiological compensation mechanisms such as dilation of blood vessels, increase of red blood cells, and increase of cerebral blood flow. When oxygen supply is insufficient, cell mitochondria produce reactive oxygen species (ROS), reactive nitrogen species (RNS), and other free radicals, which lead to oxidative stress and change DNA structure. This leads to cell damage and apoptosis [40,41,42,43]. The accumulation of free radicals in the body causes the disorder of the oxidation system or antioxidant system, leading to oxidative damage [44], which destroys the balance of the oxidation and antioxidant systems in the brain, resulting in brain damage [45,46], thus affecting cognitive processing.

Studies on animal models of chronic hypobaric hypoxia (HH) found that the degree of cognitive impairment was closely related to oxidative stress, and depended on the speed of elevation rise, altitude, and duration of residence. Hypoxia differentially affects the antioxidant status of cortex, hippocampus, and striatum [42]. Human studies have also found that high-altitude exposure is often associated with oxidative stress and is closely related to the degree of brain damage. Long-term living at high-altitude areas results in long-term imbalance between oxygen free radical formation and antioxidant defense, leading to systemic oxidative nitrification inflammatory stress and accelerating cognitive impairment in patients with chronic altitude sickness [47]. High-altitude exposure can also cause mitochondrial dysfunction in the brain, enhance oxidative stress, and increase the risk of suicide, depression, and bipolar disorder [48].

### 3.2. Cellular Mechanism

Long-term hypoxia leads to high altitude polycythemia (HAPC), manifested as hyperplasia of red blood cells, increased blood viscosity, and clinical symptoms such as dizziness, headache, and shortness of breath [49]. High altitude polycythemia affects individual’s cognitive functioning. Using the VBM (voxel-based morphometry) technique, it was found that the gray matter volume of right lingual gyrus, posterior cingulate gyrus, bilateral parahippocampus gyrus, and left inferior temporal gyrus increased in patients with high altitude polycythemia compared with subjects who do not have the disease [50]. The volume of the left anterior cingulate gyrus decreased compared to normal, which may cause some impaired visual acuity, memory, or cognitive function in patients [50]. Similarly, resting oxygen-level-dependent functional magnetic resonance imaging (BOLD-fMRI) was used to observe the brain tissue structure and function of patients with high erythrocytosis. It was found that the ReHo values of left parahippocampal gyrus and left posterior central gyrus increased, indicating that the local neuron activity in these two brain regions increased compared with normal people. However, the ReHo values of bilateral inferior temporal gyrus, right fusiform gyrus, and left middle frontal gyrus showed a downward trend, which was related to reduced cognitive functions such as amnesia [51].

High altitude polycytophysia can also affect sleep quality, manifested as difficulty in falling asleep, easy or early awakening at night, subsequently affecting cognitive function, resulting in the decline of attention span and attention transfer ability, short-term memory, attention, thinking flexibility, and other cognitive functions [52,53,54], and the lower the sleep quality, the worse the cognitive level.

In conclusion, long-term exposure to a high altitude and low-oxygen environment leads to hyperplasia of red blood cells, which leads to cumulative changes in brain structure and local fine tissue structure and function, thus negatively affecting cognitive function and reducing cognitive level.

### 3.3. Molecular Mechanism

The most fundamental cause of HAPC is chronic hypobaric hypoxia at high altitude. In order to obtain more oxygen, the human body will promote the liver and kidney to secrete a large amount of erythropoietin (EPO) through the HIF-EPO (hypoxia inducible factor-erythropoietin) pathway [55]. The EPO-EPOR (erythropoietin-erythropoietin receptor) system functions to promote red blood cell production in order to improve oxygen supply. High-altitude, low-pressure hypoxic environments can induce hypoxia-inducible factor (HIF), erythropoietin (EPO), vascular endothelial growth factor (VEGF), iron metabolism and hypoxia-induced inflammatory factors to affect erythropoiesis and then lead to HAPC, resulting in cumulative changes in brain structure and brain function, and ultimately affect cognitive function [51,55,56,57]. For example, HIF-1α may cause brain damage by promoting neuronal autophagy activation, leading to hypoxia and ischemia [58].

## 4. Retrospect and Outlook

### 4.1. Research Content

Previous studies on the effects of long-term altitude hypoxia on cognitive function have focused on basic cognitive abilities such as perception, memory, and attention, but lack of discussions on higher cognitive functions such as thinking and language. Acute exposure to altitude hypoxic environment has negative effects on thinking and language, mainly manifested as slow thinking and impaired language fluency [59,60]. Whether long-term exposure to high altitude hypoxia has a similar effect on higher cognitive function to acute hypoxia, or is different due to functional compensation, or leads to more severe impairment is unknown at present, and requires attention in future studies.

### 4.2. Research Subjects

In the past, long-term high-altitude hypoxia studies mostly took young people as the research object, and there was a sample particularity, in that most of the studies used college students as their test population. Thus, it was difficult to reflect the real situation of ordinary participants. In addition, there is a lack of research on middle-aged and elderly groups in high-altitude hypoxia environments. There is a phenomenon of premature aging among plateau residents. According to the statistics of the fourth population census of Tibet, the average life expectancy of residents on the Tibetan plateau is 58.37 years, which is about 10 years lower than that of the plains people, with the average life expectancy decreasing with the increase of altitude, and the average life expectancy decreasing by about 0.2 years for every 100 m of elevation increase. Long-term hypoxia causes damage to vital organs and accelerates aging while triggering body compensation [61]. The cognitive function of the elderly declines during the normal aging process, and whether the plateau environment accelerates the cognitive function decline of the elderly needs to be confirmed by further research.

### 4.3. Research Methodology

Existing studies mainly use cross-sectional research methods, with few longitudinal tracking reports, and few field reports on plateaus with large sample sizes [62]. Longitudinal studies can systematically and thoroughly understand the continuous process and the regularity of quantitative and qualitative changes in the action of the plateau hypoxic environment, which is helpful to determine the causal relationship. A large sample survey is conducive to the overall understanding of the impact of exposure to high-altitude hypoxic environments on the cognitive function of long-term lowlanders.

### 4.4. Research Prospects

There is still a lack of systematic on-site tracking research on the impact of the plateau environment on cognitive function. Future research needs to be careful in selecting sites in terms of the time dimension and controlling the altitude in the spatial dimension. In this way, changes of cognitive function at different altitudes and exposure times, as well as the corresponding relationship between cognition and physiological adaptation, can be more accurately revealed.

In terms of cognitive protection of the plateau environment, there are still few studies. In response to altitude hypoxia, hyperbaric oxygen interventions play an important role in the prevention and treatment of altitude sickness and acute altitude sickness. It improves sleep quality among highland migrants by effectively regulating sympathetic and vagus nerve balance [63,64]. It can also effectively improve brain injury and cognitive functions such as spatial memory and learning [65,66]. Another study has shown that hyperbaric oxygen intervention can regenerate telomere length by more than 20% and reduce senescent cells by 10% to 37%, thereby delaying aging [67]. However, there is still a lack of oxygen intervention studies for the hypoxic plateau environment, and future studies on hyperbaric oxygen intervention, diffuse oxygen, nasal oxygen intervention, etc., will help determine the altitude oxygen dosage standard, effectively solve the problem of plateau hypoxia, and provide an empirical basis for plateau residents to nourish and protect their brains.

In addition, altitude cognitive impairment can be improved by modulating gut microbiota. The gut–brain axis is a system composed of the brain and the gut in the human body. The gut and the brain communicate through hormones and neural information to jointly regulate emotional responses, metabolism, immune system, brain development and health [68,69]. The gut microbiota is closely related to cognitive function, and probiotic intervention can modulate the gut microbiota, thereby improving cognitive function [70]. High altitude hypobaric and hypoxic environments have a significant impact on the body’s cognitive function and intestinal flora [71,72], and microbiological techniques can be used to regulate intestinal flora and affect physiological and cognitive functions of the patients, making them more adaptable to the high-altitude hypoxic environment.

In the future, a set of effective guidelines for healthy lifestyles for people at high altitudes can be explored in combination with high-altitude oxygen use and special diet research.

## 5. Conclusions

Long-term exposure to a high altitude hypoxic environment affects cognitive function, which is manifested in features such as attention, memory ability, and inhibitory control. In this paper, the underlying mechanism is summarized, and the neural, stress, cellular, and molecular mechanisms by which long-term high-altitude hypoxia exposure affects cognitive function are discussed. Future research needs to continue to expand the scope of cognitive function research, and large-scale follow-up studies need to be conducted. It is also necessary to strengthen the research on brain nourishing and brain protection at high altitudes, and explore the adaptation methods for high-altitude hypoxic environments. Finally, for those interested in entering high altitudes for tourism, work, or to live, it remains necessary to be cautious and possibly prepare by means of preconditioning or medication.

## Data Availability

Data materials can be obtained by contacting the corresponding author.
